# Effects of* Astragalus* Polysaccharides on Dysfunction of Mitochondrial Dynamics Induced by Oxidative Stress

**DOI:** 10.1155/2016/9573291

**Published:** 2016-01-11

**Authors:** Yan-Feng Huang, Lu Lu, Da-Jian Zhu, Ming Wang, Yi Yin, De-Xiu Chen, Lian-Bo Wei

**Affiliations:** ^1^School of Traditional Chinese Medicine, Southern Medical University, Guangzhou 510515, China; ^2^Department of Gastrointestinal Surgery, Shunde First People's Hospital, Southern Medical University, Guangdong 528300, China; ^3^Department of Traditional Chinese Medicine, ZhuJiang Hospital, Southern Medical University, Guangzhou 510280, China; ^4^Department of Nephrology, Southern Medical University TCM-Integrated Hospital, Guangzhou 510515, China

## Abstract

This paper studied the chronic fatigue induced by excessive exercise and the restoration effects of* Astragalus* polysaccharides (APS) on mitochondria. In vivo, we found that excessive exercise could cause oxidative stress statue which led to morphological and functional changes of mitochondria. The changes, including imbalance between mitochondria fusion-fission processes, activation of mitophagy, and decrease of PGC-1*α* expression, could be restored by APS. We further confirmed in vitro, and what is more, we found that APS may ameliorate mitochondrial dysfunction through Sirt1 pathway. Based on the results, we may figure out part of the molecular mechanism of mitochondrial amelioration by APS.

## 1. Introduction

Fatigue is defined as a lack of energy, mental or physical tiredness, diminished endurance, and the need for prolonged recovery after physical activity. Physical fatigue can be due to the action of the muscles, but fatigue can also be a hallmark symptom of mitochondrial disease [1]. Excessive exercise, which differs from moderate, regular training, could cause the accumulation of an excess of reactive free radicals, which ultimately leads to tissue damage and muscle dysfunction.

Mitochondria play a crucial role in energy production. Usually, mitochondrial dysfunction can be reversed through various protective mechanisms (e.g., fission and fusion); however, if the mechanisms are altered, mitochondrial dysfunction can cause multifarious diseases. Alterations in energy metabolism have been shown to contribute to fatigue. Dysfunction in mitochondrial structure, function (mitochondrial enzymes and oxidative/nitrosative stress), and energy metabolism (ATP production and fatty acid metabolism) as well as the immune response and genetics have been investigated as potential contributors to fatigue [2].

*Astragalus membranaceus* (Leguminosae) is a popular “Qi-tonifying” herb and is often used in Chinese traditional medicinal formulas for Qi (vital energy) deficiency, which is characterized by fatigue, limb weakness, lack of appetite, and dizziness.* Astragalus membranaceus* has been shown to enhance endurance in mice, and its use as an ergogenic and antifatigue agent has been suggested [3].* Astragalus* polysaccharides (APS) are one of the main bioactive components extracted from* Astragalus membranaceus*. Previous studies have shown that APS inhibits both the mitochondrial injury caused by the continuous production of free radicals and selective oxidative damage [4], which is commonly associated with fatigue. APS is thought to protect mitochondrial function by scavenging reactive oxygen species (ROS) and inhibiting the opening of a mitochondrial permeability transition pore [5]. However, the underlying mechanisms have not been clarified. In this study, we established a chronic fatigue model based excessive exercise in mice and evaluated signalling pathways and gene and mRNA levels related to mitochondrial biogenesis, proliferation, and function to demonstrate the protective effect of APS on mitochondrial dysfunction.

## 2. Materials and Methods

### 2.1. Extraction and Purification of APS

*Astragalus membranaceus* was purchased from Guangzhou Qingping Medicine Market (Guangzhou, China) and identified by the Department of Authentication of Chinese Medicine, Chinese Traditional Medicine (Wuhan, China).

APS was prepared using optimized direct water decoction techniques as previously described [6] and purified by the Sevag method and chromatography using a Sephadex G-200 column. The polysaccharide content was determined to be 95.25% by the phenol-sulfuric acid method. Then, the chemical constituents (monosaccharides and polysaccharides fractions) were identified by gas chromatography analysis (shown in Supplementary Figure 1 and Table 1 (see Supplementary Material available online at http://dx.doi.org/10.1155/2016/9573291)). Endospecy assay showed that the APS samples did not contain a detectable level of endotoxin (<0.10 endotoxin units/mL).

### 2.2. Experimental Animals and Experimental Design

Specific pathogen-free BALB/c male mice (8 weeks old, 17~20 g) were obtained from the Experimental Animal Center of Southern Medical University, China (Approval number SCXK (Yue) 2011-0015). BALB/c mice are the most suitable strain for the evaluation of swimming endurance capacity [7] because they are resistant to diet-induced obesity [8]; this minimizes the potential confounding effects of body fat accumulation, which increases floating ability in water, on endurance capacity. The mice were housed 5 per cage in a room maintained at 23°C ± 2°C with a 12 h light/dark cycle (lights on from 6:00 am to 6:00 pm) and were provided ad libitum access to food and water. The mice were treated in accordance with the guidelines for the Care and Use of Laboratory Animals formulated by the Ministry of Science and Technology of China, and all experimental procedures were approved by the Animal Ethics Committee of Southern Medical University. All efforts were made to minimize the number of animals used and their suffering.

The mice were allowed to acclimate for 1 week before the experiments were begun. During this period, the mice were subjected to two practice swimming sessions (10 min per time), and those that could not learn to swim were screened out. Then, 40 mice were chosen and randomly divided into four groups (*n* = 10/group): (i) sedentary (SED) group: the mice were allowed free access to a standard rodent diet and treated with saline solution; (ii) sedentary with APS supplementation (SED + APS) group: the mice were allowed free access to a standard rodent diet and treated with 100 mg/kg/day of APS in saline solution; (iii) endurance exercise (EXE) group: the mice were allowed free access to a standard rodent diet and treated with saline solution; (iv) endurance exercise with APS supplementation (EXE + APS) group: the mice were allowed free access to a standard rodent diet and treated with 100 mg/kg/day of APS in saline solution. Each mouse was labelled via the application of picric acid to its back.

APS was dissolved in 2.0 mL of saline solution, and mice in the SED group and EXE group received the same volume of saline solution. The treatments were administered orally (8:00 am) by gavage using an atraumatic feeding needle, once per day for 28 consecutive days.

### 2.3. Endurance Exercise Procedure

All mice were monitored for changes in body weight during the endurance exercise procedure. The mice in the EXE and EXE + APS groups were subject to endurance exercise 4 days per week (duration: 4 weeks). The procedure used in this experiment was similar to that described by Porsolt et al. [9]. The mice were individually placed in a swimming pool (height: 30 cm, diameter: 25 cm) in which they could only support themselves by touching the bottom with their feet (at 25°C ± 1°C). A tin wire (approximately 10% of body weight, weighing 1 g, 1.5 g, 2 g, 2.5 g, 3 g, 3.5 g, or 4 g) was loaded onto the base of the tail of each mouse. The swimming period was regarded as the time the mouse spent floating, struggling, and making movements in the water until its strength was exhausted. Exhaustion was defined as the inability to rise to the surface of water to breathe within a 7 s period. At the end of the session, the mice were removed from the water, dried with a paper towel, relabelled with picric acid, and placed back in their home cages. The water in the container was drained after each session. To prevent adaptation, the exercise onset time was chosen randomly each day (at least 1 h after the gavage). After 4 weeks of exercise, endurance capacity was measured on the 29th day. All of the mice were subjected to a weight-loaded swimming test (WLST), the procedure of which was the same as that described above. The swimming time to exhaustion was used as the index of the forced swimming capacity.

### 2.4. Forelimb Grip Strength

A low-force testing system (Model-RX-5; Aikoh Engineering, Nagoya, Japan) was used to measure the forelimb grip strength of all of the mice. The amount of tensile force exerted by each mouse was measured using a force transducer equipped with a metal bar (2 mm in diameter and 7.5 cm in length). The detailed procedure was the same as that described previously [10]. The test was performed on the last day of each week (at least 1 h after the gavage and including 2 h of access to food and water if endurance exercise was performed on the same day). The maximal force (grams) recorded was used as an indicator of absolute grip strength.

### 2.5. Sample Preparation

All mice were sacrificed under ether anaesthesia via intraperitoneal injection of ketamine (80 mg/kg) and xylazine (4 mg/kg) 60 min after the last WLST. Blood samples from the mice were collected in tubes by heart puncture. Skeletal muscles (gastrocnemius muscle of both hind legs) were collected rapidly under standard conditions. Serum was separated by centrifugation at 3,000 rpm at 4°C for 10 min. The skeletal muscles were isolated immediately and then stored at −80°C until further use.

### 2.6. Oxidative Stress-Related Biochemical Analysis

Blood levels of superoxide dismutase (SOD), malondialdehyde (MDA), glutathione peroxidase (GPx), and lactate dehydrogenase (LDH) were determined using commercially available kits from the Jiancheng Bioengineering Institute (Nanjing, China).

Portions of the skeletal muscle samples were homogenized in ice-cold buffer (0.25 M sucrose, 10 mM Tris-HCl, and 0.25 Mm phenylmethylsulphonyl fluoride; pH 7.4), and a portion of the homogenate was immediately measured for MDA levels. Another portion of the homogenate was centrifuged at 15,000 g for 30 min at 4°C, and the supernatant was decanted and assayed for SOD and GPx activity. SOD and GPx activity as well as MDA levels were determined according to the recommended procedures provided by the commercial kits (Jiancheng Bioengineering Institute, Nanjing, China).

### 2.7. Cell Culture and Differentiation

C2C12 myoblasts were purchased from the Type Culture Collection of the Chinese Academy of Sciences, Shanghai, China. The cells were cultured in high-glucose DMEM containing 10% foetal bovine serum, 2 mM glutamine, 100 units/mL penicillin, and 100 *μ*g/mL streptomycin. The cells were maintained at 37°C under 5% CO_2_ (v/v) in a humidified incubator. The C2C12 myoblasts were switched to differentiation medium (DMEM containing 2% horse serum) when 80% confluent. The differentiation medium was exchanged every 2 days for 6 days before experimental manipulation.

### 2.8. Treatments of Cells

According to our previous study, APS significantly stimulated C2C12 myotube proliferation at a concentration of 0.2 mg/mL, and mitochondrial activity was improved in 24 h [11]. In this study, C2C12 myotubes were pretreated with APS (0.2 mg/mL) for 24 h and then treated with or without tert-butylhydroperoxide (t-BHP) (100 *μ*M, some C2C12 myotubes were also treated with 0.2 mg/mL APS during this period, based on our preliminary experiments) for another 24 h. The cells were also treated with or without EX-527 (2 *μ*M) for 46 h following the addition of APS for 2 h. All treatments were performed in complete culture medium to avoid the induction of autophagy through the serum starvation pathway.

### 2.9. Measurement of ROS Production

Differentiated C2C12 cells (1.0 × 105 cells/well) in a 96-well plate were treated in the same manner described above. ROS generation was measured by incubating the cells with 5 *μ*M 2′,7-dichlorodihydrofluorescein diacetate (DCFH2-DA, Sigma) at 37°C for 30 min. Fluorescence, which is directly related to ROS production, was measured with a microplate reader at excitation and emission wavelengths of 485 nm and 530 nm, respectively.

### 2.10. Measurement of Mitochondrial Membrane Potential (ΔΨm)

The ΔΨm was assessed in differentiated myoblasts using the fluorescent, lipophilic, and cationic probe 5,5′,6,6′-tetrachloro-1,1′,3,3′-tetraethylbenzimidazolylcarbocyanine iodide (JC-1) according to the manufacturer's directions. For quantitative fluorescence measurements, the cells were rinsed three times with PBS after JC-1 staining and scanned with a microplate reader (Fluoroskan Ascent; Thermo Fisher Scientific, Waltham, MA, USA) at an excitation wavelength of 488 nm and emission wavelengths of 535 nm (mitochondrial JC-1 monomers) and 590 nm (mitochondrial JC-1 aggregates). Each well was evaluated by measuring the intensity of each of 25 squares (1 mm^2^ in area) arranged in a 5 × 5 rectangular array. The ΔΨm of the C2C12 myotubes in each treatment group was determined from the ratio of red fluorescence (i.e., aggregates) to green fluorescence (i.e., monomers).

### 2.11. Quantitative RT-PCR Analyses

Total RNA was extracted from the skeletal muscle samples and the treated cells using the RNAiso Plus reagent (Takara, number 9108) according to the supplier's protocol and was quantified using a spectrophotometer set at 260 nm. cDNA was synthesized using an RT reagent kit (TaKaRa, number RR047A) at 37°C for 15 min and at 85°C for 5 s. Quantitative RT-PCR was performed using the SYBR PrimeScript RT-PCR Kit (Takara, number RR820A) on a Stratagene MX3005P QRT-PCR system (Agilent Technologies, Santa Clara, USA). The primers used in the QRT-PCR reactions are shown in Table 1. The thermal cycles for PCR amplification were carried out with an initial denaturation at 95°C for 5 min, followed by 45 cycles of denaturation (10 s, 95°C), annealing (10 s, 60°C for Drp-1; 60.5°C for Mfn-1, Opa-1, and GAPDH; 61°C for Mfn-2; and 62°C for Fis-1), and extension (12 s, 72°C).

### 2.12. Western Blotting Analyses

The skeletal muscle tissues were lysed with liquid nitrogen and RIPA lysis buffer (Beyotime, Jiangsu, China). The lysates were homogenized, and the homogenates were centrifuged at 13,000 g for 15 min at 4°C. Proteins were isolated from cultured cells using RIPA lysis buffer and by following the same protocol. The supernatants were collected, and protein concentrations were determined with a BCA Protein Assay kit (Pierce, number 23225). Equal aliquots (30 *μ*g) of the protein samples were applied to 10% SDS-PAGE gels, transferred to polyvinylidene fluoride (PVDF) membranes, and blocked with 5% skim milk TBST (Tris-buffered Saline Tween-20) buffer. The membranes were incubated with primary antibodies including anti-PGC-1, anti-MnSOD, anti-p53, and anti-GAPDH (1 : 1,000; Santa Cruz Biotechnology) as well as anti-Atg7, anti-p62, and anti-LC3 (1 : 1,000; Cell Signaling) at 4°C overnight. Then, the membranes were incubated with anti-rabbit or anti-mouse antibodies at room temperature for 1 h. The protein bands were captured and documented through a CCD system (Image Station 2000MM, Kodak, Rochester, NY, USA) or a gel image analysis system (ChemiDox XRS, Bio-Rad, USA).

### 2.13. Laser Scanning Confocal Microscopy

After the experimental interventions, the cells were loaded with MitoTracker Green (MTG, 0.5 *μ*M) for 60 min at 37°C in culture medium. During the last 20 min of staining with MTG, the cells were also loaded with LysoTracker Red (LTR, 0.5 *μ*M) under identical conditions. The MTG and LTR fluorescence was monitored with a Nikon Confocal Microscope using a 63x oil, 1.4 NA objective lens. Probe excitation and fluorescence emission were also measured as previously described [12].

### 2.14. Statistical Analyses

The quantitative data are presented as the mean ± standard deviation (SD). The variance of the data was first analysed using a homogeneity test. If the data met the assumption of homoscedasticity, the significance of differences in the means was determined by one-way ANOVA followed by an LSD *t*-test for multigroup comparisons. Otherwise, significance was determined by Tamhane's T2 test. All statistical analyses were performed using SPSS 13.0 statistical software (SPSS, Chicago, IL, USA). Pearson correlation and linear regression were used to assess correlations between continuous variables. A *P* value < 0.05 was considered statistically significant.

## 3. Results

### 3.1. Effects of Excessive Exercise and APS Supplementation on Physical Performance

**Figure 1:**
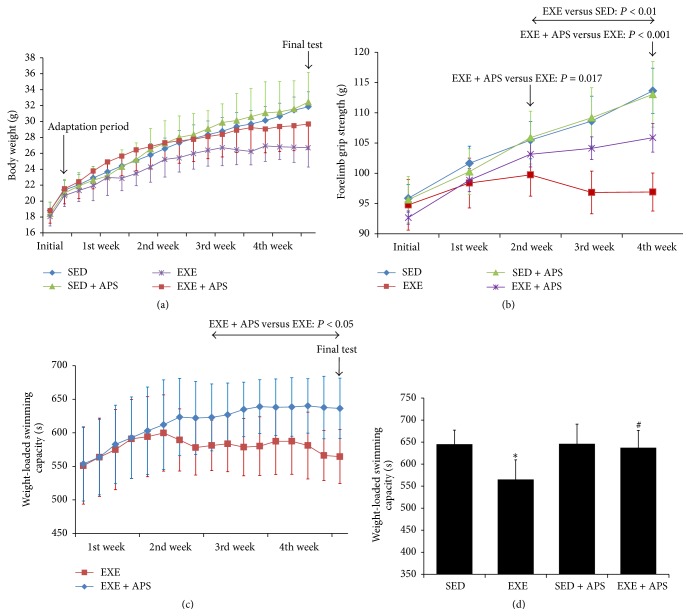
Effects of EXE and APS supplementation on physical performance. (a) Changes of body weight during the whole period. (b) Effects of APS on forelimb grip strength in BALB/c mice. (c) Changes of weight-loaded swimming capacity in mice of EXE and EXE + APS groups. (d) Effects of APS on weight-loaded swimming capacity in BALB/c mice on the final test. Values are means ± SEM; ^*∗*^ *P* < 0.05 versus SED group; ^#^ *P* < 0.05 versus EXE group.

Body weight was recorded before every endurance exercise and the final WLST. The body weight data are shown in Figure 1(a). There were no significant differences in body weight among the four groups (*P* > 0.05) during the experimental period. Both exercise and APS supplementation had no obvious effect on body weight.

There were no differences in initial forelimb grip strength between the SED, EXE, SED + APS, and EXE + APS groups. As shown in Figure 1(b), after 2 weeks of intervention, the forelimb grip strength of the mice in the EXE group was lower than that of those in the SED group (*P* < 0.01), and the differences persisted until the end of the experiment (*P* < 0.001). Notably, the strength of the mice in the EXE + APS group was higher than that of those in the EXE group in the 2nd week and the 4th week (*P* = 0.017 and *P* < 0.001). In contrast, there were no significant differences between the SED and SED + APS groups throughout the whole period. We found that the grip strength of mice that received APS supplementation was significantly increased compared with that of the EXE mice but not with that of the SED mice.

During the endurance exercise procedure, the swimming time to exhaustion of the EXE + APS group appeared longer than that of the EXE group, and the results reached significance during the 3rd week (*P* < 0.05). According to the data collected from all of the mice during the weight-loaded swimming test on the 29th day, excessive exercise tended to reduce endurance capacity (*P* < 0.05), and APS supplementation was sufficient to improve it (*P* < 0.05), without affecting the endurance of sedentary mice (*P* > 0.05), as shown in Figure 1(c).

### 3.2. Effect of APS on Oxidative Stress-Related Biochemical Parameters in Mice

**Figure 2:**
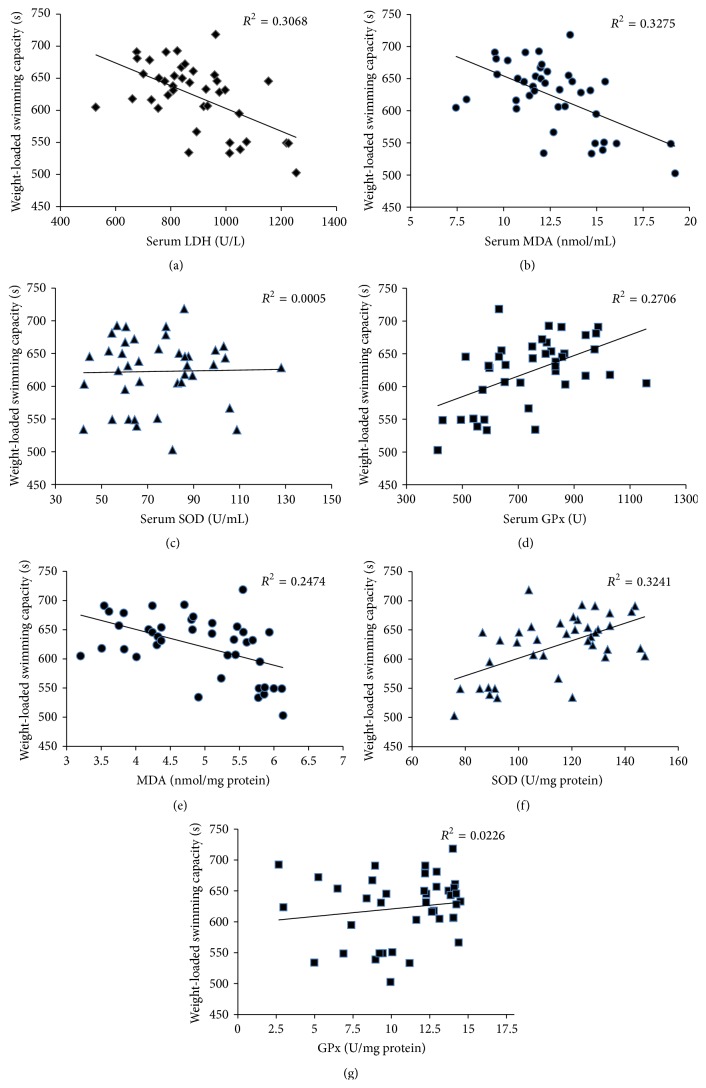
Correlation between endurance capacity and biochemical parameters of oxidative stress in mice.

Pearson correlation was used to analyse the influence of oxidative stress parameters on physical performance. There was a moderate negative correlation between endurance capacity and serum LDH and MDA levels (LDH: *r* = −0.554, *P* = 0.0002; MDA: *r* = −0.572; *P* = 0.0001). A similar relationship was also observed between the MDA level in muscle tissue and endurance capacity (*r* = −0.497, *P* = 0.001). Antioxidant defence status was also positively correlated (serum GPx level: *r* = 0.52, *P* = 0.001; SOD level in muscle tissue: *r* = 0.569, *P* = 0.0001) with endurance capacity (Figure 2).

**Figure 3:**
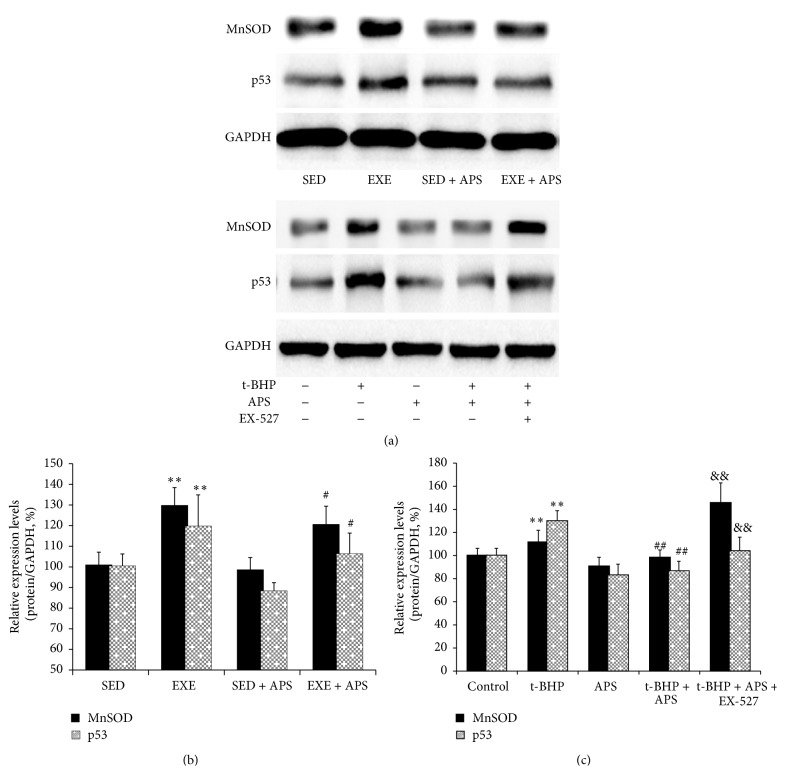
Protein levels of p53 and MnSOD. (a) Western blot images. (b) Statistical results of proteins in vivo. (c) Statistical results of proteins in vitro. Values are means ± SEM; ^*∗∗*^ *P* < 0.01 versus SED or control group; ^#^ *P* < 0.05 versus EXE or t-BHP group; ^##^ *P* < 0.01 versus EXE or t-BHP group; ^&&^ *P* < 0.01 versus t-BHP + APS group.

Moreover, we examined proteins indicative of oxidative status and found that both MnSOD and p53 were upregulated by exercise (Figures 3(a) and 3(b)) in vivo. Both were upregulated by excessive exercise, and the increase in both could be inhibited by APS supplementation (Figure 3).

### 3.3. Effect of APS on Mitochondrial Biogenesis and Autophagy Activation In Vivo

**Figure 4:**
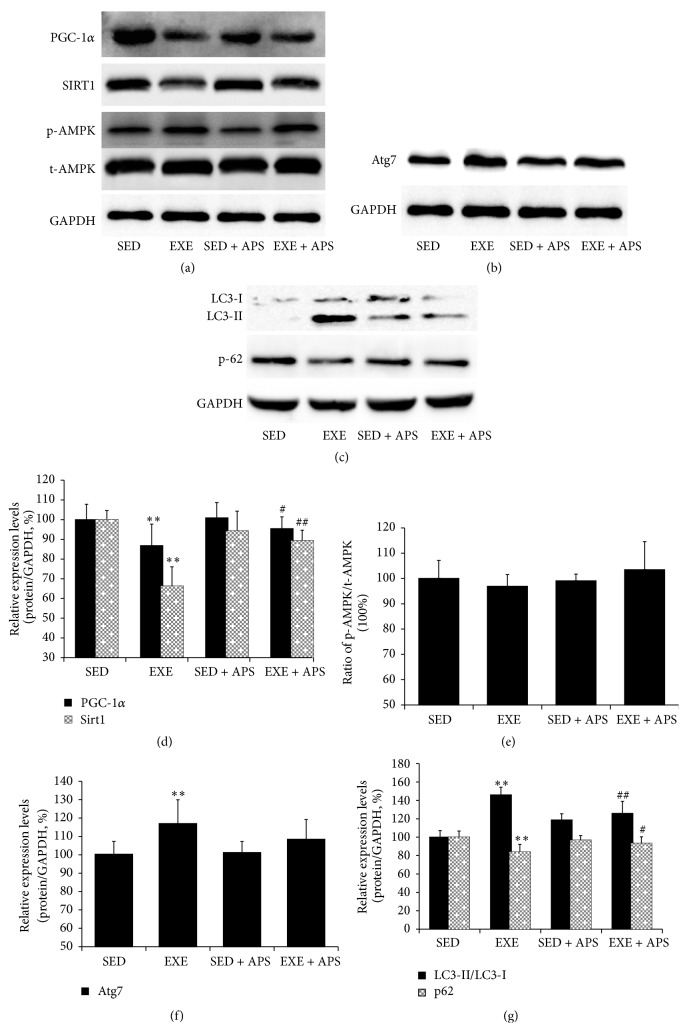
Effects of APS on mitochondrial biogenesis and autophagy activation in vivo. ((a), (d), (e)) Protein levels of PGC-1*α*, Sirt1, and AMPK ((a) Western blot images; (d), (e) statistical results); ((b), (f)) protein levels of Atg7 ((b) Western blot images; (f) statistical results); ((c), (g)) protein levels of LC3-I, LC3-II, and p62 ((c) Western blot images; (g) statistical results). Values are means ± SEM; ^*∗∗*^ *P* < 0.01 versus SED group; ^#^ *P* < 0.05 versus EXE group; ^##^ *P* < 0.01 versus EXE group.

Western blotting showed that excessive exercise decreased PGC-1*α* (a type of transcriptional coactivator essential to mitochondrial biosynthesis) expression (*P* < 0.05) and that the expression could be restored by APS supplementation (Figures 4(a) and 4(d)). These findings were similar to the physical performance results of the mice. The level of Sirt1 expression significantly was decreased by exercise (*P* < 0.05), but the decrease could be ameliorated by APS supplementation (*P* < 0.05) (Figures 4(a) and 4(d)). In contrast, no significant differences in AMPK expression were observed among the four groups (*P* > 0.05) (Figures 4(a) and 4(e)). Unexpectedly, expression of the autophagy-related proteins Atg7 and LC3-II was strongly induced, whereas the expression of p62 was decreased. All of these changes could be ameliorated by APS supplementation (Figures 4(b), 4(c), 4(f), and 4(g)).

### 3.4. Effect of APS on Mitochondrial Fusion/Fission In Vivo

As shown in Table 2, the expression of mitochondrial fusion-related genes (Mnf-1, Mnf-2, and Opa-1) was lower in skeletal muscle samples from the EXE group; the expression of the fission-related gene Drp-1 was also lower in the mice that were subjected to the exercise procedure, but the expression of Fis-1 did not change significantly. The changes in Mnf-1, Mnf-2, and Drp-1 expression could be prevented if the EXE mice received APS supplementation.

### 3.5. Effect of APS on Mitochondrial Quality Control (QC) In Vitro

**Figure 5:**
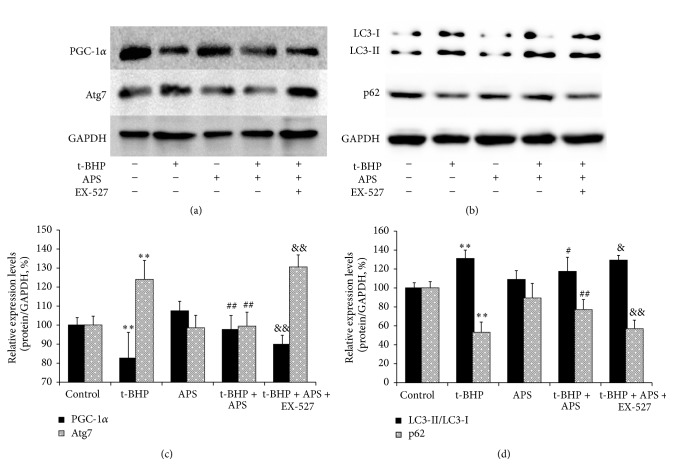
Effects of APS on mitochondrial biogenesis and autophagy activation in vitro. ((a), (c)) Protein levels of PGC-1*α* and Atg7 ((a) Western blot images; (c) statistical results). ((b), (d)) Protein levels of LC3-I, LC3-II, and p62 ((b) Western blot images; (d) statistical results). Values are means ± SEM; ^*∗∗*^ *P* < 0.01 versus control group; ^#^ *P* < 0.05 versus t-BHP group; ^##^ *P* < 0.01 versus t-BHP group; ^&^ *P* < 0.05 versus t-BHP + APS group, ^&&^ *P* < 0.01 versus t-BHP + APS group.

We hypothesized that the induction of oxidative stress in the skeletal muscle due to exercise might activate autophagy, which could have a negative impact on mitochondrial biosynthesis. To test this hypothesis, we used the C2C12 mouse cell line and applied t-BHP to simulate the changes in skeletal muscle. As shown in Figure 5, PGC-1*α* levels were significantly decreased in the t-BHP group compared to the control group, and MnSOD and p53 were upregulated (Figures 3(a) and 3(c)). The autophagy markers Atg7 and LC3 increased, whereas p62 decreased (Figure 5). On the other hand, APS supplementation could restore the levels of PGC-1*α* as well as of the autophagy markers LC3, p62, and Atg7. It could also inhibit the increase of MnSOD and p53. These APS-related changes were effectively eliminated by the Sirt1 antagonist EX-527.

**Figure 6:**
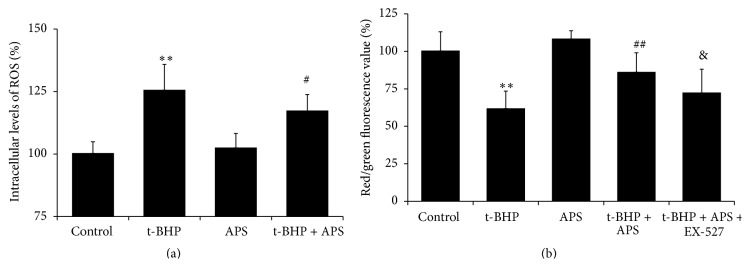
(a) Measurement of ROS production in vitro; (b) mitochondrial membrane potential (ΔΨm) in vitro. Values are means ± SEM; ^*∗∗*^ *P* < 0.01 versus SED or control group; ^#^ *P* < 0.05 versus EXE or t-BHP group; ^##^ *P* < 0.01 versus EXE or t-BHP group; ^&^ *P* < 0.05 versus t-BHP + APS group.

In vitro, MnSOD and p53 were upregulated by t-BHP-induced oxidative stress, although these increases could be inhibited by APS supplementation (Figures 3(a) and 3(c)). As shown in Figure 6(a), ROS levels in C2C12 myotubes were increased by exposure to t-BHP, and APS treatment significantly inhibited the generation of ROS. We also used JC-1 to quantify the ΔΨm, which can reflect mPTP opening. The ΔΨm is determined based on changes in JC-1 fluorescence. As shown in Figure 6(b), T-BHP reduced the ratio of red fluorescence (i.e., aggregates) to green fluorescence (i.e., monomers) (*P* < 0.01 compared to the control group), and this reduction could be prevented by APS supplementation (*P* < 0.001). Furthermore, this effect of APS could be eliminated by the Sirt1 antagonist EX-527 (*P* = 0.04).

The fusion/fission genes were also measured in vitro. T-BHP led to a decrease in Mnf-1 and Mnf-2 gene expression, and this decrease could be prevented by APS treatment. However, the intervention had no statistically significant effects on the expression of the other three genes (Table 3).

**Figure 7:**
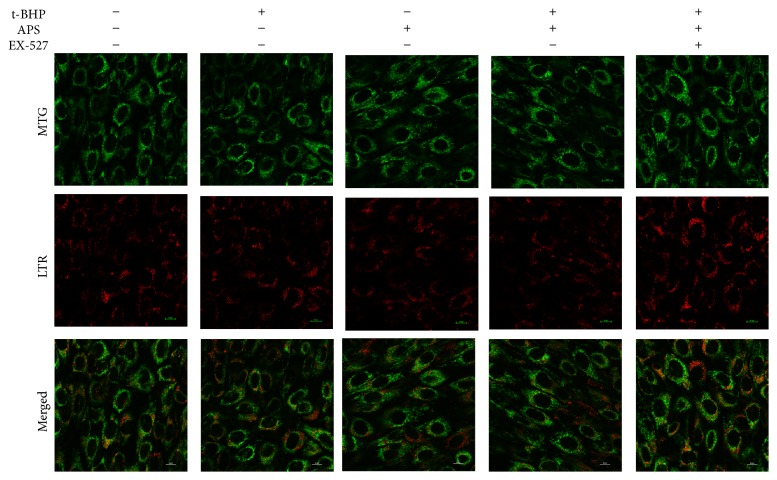
Images of fluorescence monitored with a Nikon Confocal Microscope using 63x oil 147 NA objective lens. MTG: MitoTracker Green; LTR: LysoTracker Red.

To integrate all of the mitochondrial biogenesis, proliferation, and maturation results in vivo, we performed laser scanning confocal microscopy.  Figure 7 shows images of C2C12 myotubes that were colabelled with MTG and LTR. The APS-treated C2C12 cells exhibited a more stacked mitochondrial reticulum that also showed more MTG fluorescence (mitochondria) but less LTR fluorescence (lysosomes). The colocalization of MTG and LTR was also decreased in the APS + t-BHP-treated cells compared to the t-BHP-treated ones (the APS + t-BHP group versus the t-BHP group). When EX-527 was added, similar changes (a decrease in MTG fluorescence and an increase in LTR fluorescence) were observed.

## 4. Discussion

Mitochondria are multifunctional organelles found in eukaryotic cells that are responsible for most of the energy generation in human and animal cells. There are two key cellular mechanisms involved in the maintenance of mitochondrial function: mitochondrial turnover and the fusion-fission process [13]. Mitochondria are continuously turned over through the complementary processes of mitochondrial biogenesis (mitogenesis) and mitochondrial autophagy (mitophagy), which form the backbone of the mitochondrial QC process [14].

Exercise programmes vary in terms of the duration, frequency, speed, and type of exercise. Training frequency has been reported to directly affect the capacity to perform endurance exercise [15]. Skeletal muscle is one of the most critical tissues involved in the development of exercise tolerance, as it responds differently to exercise of various intensities [16–18]. We considered the excessive exercise required by our training protocol to be a model of chronic fatigue. Based on the regression of the biochemical parameters of oxidative stress with exercise capacity, we hypothesized that excessive exercise would induce dramatically higher ROS production, which would then decrease exercise capacity. Increased MnSOD and p53 expression was also observed. With respect to the function of the organelle, the JC-1 fluorescence results suggested that oxidative stress had a negative effect on the ΔΨm. We hypothesized that excess exercise dramatically increased ROS production, which would inhibit mitochondrial biosynthesis, trigger mitophagy, and disrupt the balance of the mitochondrial fusion-fission process.

Mitochondria are a major source of ROS [19], and mitochondrial perturbations caused by oxidative stress have been intimately linked to autophagy/mitophagy. The removal of damaged mitochondria by mitophagy is critical for the maintenance of mitochondrial QC and cellular homeostasis [20]. Knockout of Atg7 (a crucial autophagy gene) in mice resulted in profound muscle atrophy and an age-dependent decrease in muscle force, and Atg7 null muscles showed the accumulation of abnormal mitochondria [21]. Moreover, knocking down the critical gene LC3 by RNAi partially prevented muscle loss [22]. Lo Verso et al. reported that autophagy could prevent the accumulation of dysfunctional mitochondria during damaging muscle contraction [23]. In our study, expression of the autophagy markers Atg7 and LC3 was strongly induced by excessive exercise, whereas the expression of p62 was decreased. Thus, we concluded that excess exercise may trigger mitophagy. However, if damaged mitochondria cannot be discarded through mitoptosis, cell dysfunction could result due to the increase in ROS production [24]. Proliferation and ROS-related signalling pathways could also be activated [25].

Peroxisome proliferator-activated receptor *γ* coactivator-1 (PGC-1) is a superfamily of transcriptional coactivators that are important precursors to mitochondrial biosynthesis. To increase muscle mitochondrial content, peroxisome proliferator-activated receptor *γ* coactivator-1*α* (PGC-1*α*) must be activated, along with several other intracellular signalling molecules. Low levels of PGC-1*α* have previously been linked to obesity, diabetes, and several other metabolic disorders [26, 27]. Based on our results, we hypothesized that oxidative stress might be responsible for the decrease in the levels of PGC-1*α* in the skeletal muscle of the mice subjected to excessive exercise.

Mitochondrial dynamics are regulated by coordinated fusion and fission processes that are carried out through complex molecular machinery [28], and derangements in fusion-fission have been proposed as a mechanism underlying the formation of dysfunctional aberrant mitochondria [29]. Defective mitochondrial fusion has been shown to result from the downregulation of Mfn-1, Mfn-2, and Opa-1 [30]. The mRNA expression of these fusion-related genes (Mfn-1, Mfn-2, and Opa-1) was decreased in the EXE group. In contrast, the expression of fission-related genes (Drp-1) increased, whereas Fis-1 gene expression did not change significantly. Similar Mfn-1 and Mfn-2 expression results were also observed in vitro. These results could be explained by the fact that Drp-1 is a cytoplasmic protein and acts as a ligand, whereas Fis-1 is a mitochondrial protein and acts as a receptor [28]. To summarize, in the mice subjected to excess exercise, fusion was inhibited while fission was stimulated. Previous studies also reported that increased muscle expression of PGC-1*α* could prevent the reduction in fusion protein expression (Mfn-1, Mfn-2, and Opa-1) [31] and that PGC-1*α* could stimulate Mfn-2 expression [32].

We demonstrated that APS could improve the exercise capacity of mice undergoing oxidative stress. Similar to previous reports, APS could protect mitochondria by scavenging ROS, inhibiting mitochondrial swelling, increasing the activity of antioxidant enzymes, and ameliorating mitochondrial dysfunction, which ultimately improved energy metabolism [4]. In this study, we tried to determine the molecular mechanism of APS-mediated amelioration of mitochondrial dysfunction.

In vivo, we detected the expression of AMPK and Sirt1, which play critical roles in PGC-1*α* induction [33]. We found that the low level of Sirt1 expression caused by excessive exercise could be prevented by APS supplementation. Some authors previously reported that the coordination of Sirt1 and PGC-1 expression/activity is important for the induction of genes related to mitochondrial biogenesis [34]. Our study demonstrated that APS elevated both Sirt1 and PGC-1*α* protein expression in skeletal muscle suffering oxidative stress but did not affect AMPK expression. Sirt1 regulates mitochondrial biogenesis and is important for the maintenance of muscle mitochondrial content and function [34]. We hypothesized that APS ameliorated mitochondrial dysfunction through the Sirt1 pathway. In vitro, we found that EX-527 pretreatment notably inhibited the APS supplementation-induced amelioration of mitochondrial dysfunction, consistent with the PGC-1*α* expression results.

In our study, autophagy induced by oxidants could be prevented by APS, and the effects of APS could be inhibited by EX-527. This finding may be inconsistent with the idea that Sirt1 overexpression induces autophagy and that stimulators of autophagy indirectly activate Sirt1 through AMPK to trigger autophagy [35]. We hypothesized that it might not be sufficient to trigger autophagy through the AMPK-SIRT1 signalling pathway because of the low level of Sirt1 expression that was caused by the oxidative stress and because of the absence of significant differences in AMPK levels among the four groups.

Moreover, the qRT-PCR results indicated that APS supplementation promoted mitochondrial fusion and inhibited fission. Some studies have demonstrated links between mitochondrial dynamics and mitochondrial physiology. For example, the activation of mitochondrial fusion increases the ΔΨm, oxygen consumption, and mtDNA replication [28], and aberrant mitochondrial fission results in impaired mitochondrial recruitment localization [24], which is consistent with our results.

Increased mitochondrial fusion has been shown to prevent lysosome-mediated mitoptosis, which contributes to the maintenance of an incorrect mitochondrial pool as well as increased oxidative stress and mitochondrial biogenesis [36]. To integrate the results, we performed laser scanning confocal microscopy and coloaded C2C12 cells with MTG and LTR. The results showed that mitochondria were accumulating in APS-treated cells through an increase in fusion and a decrease in fission that were evident not only in mRNA expression changes but also morphologically.

## 5. Conclusion

Mitochondrial dysfunction and the morphological changes induced by oxidative stress could be restored by APS supplementation. The molecular mechanism underlying the effect of APS may involve the synergistic interactions between mitophagy and mitochondrial fusion for mitochondrial QC.

## Supplementary Material

Figure S1 shows the result of monosaccharide analysis of Astragalus polysaccharides by Gas Chromatography (GC).
Table S1 shows the result of proportional analysis of monosaccharide composition in Astragalus polysaccharides.

## Figures and Tables

**Table 1:** Primers and conditions used for RT-PCR.

Gene	Forward primer (5′-3′), reverse primer (5′-3′)	*T*-An. (°C)	Gene	Forward primer (5′-3′), reverse primer (5′-3′)	*T*-An. (°C)
Mfn-1	ATGGCAGAAACGGTATCTCCA CTCGGATGCTATTCGATCAAGTT	60.5	Drp-1	CAGGAATTGTTACGGTTCCCTAA CCTGAATTAACTTGTCCCGTGA	60

Mfn-2	CTGGGGACCGGATCTTCTTC CTGCCTCTCGAAATTCTGAAACT	61	Fis-1	TGTCCAAGAGCACGCAATTTG CCTCGCACATACTTTAGAGCCTT	62

Opa-1	TGGAAAATGGTTCGAGAGTCAG CATTCCGTCTCTAGGTTAAAGCG	60.5	GAPDH	TGGATTTGGACGCATTGGTC TTTGCACTGGTACGTGTTGAT	60.5

*T*-An.: annealing temperature; Mfn-1: mitofusin 1; Mfn-2: mitofusin 2; Opa-1: optic atrophy 1; Drp-1: dynamin-related protein q; Fis-1: mitochondrial fission 1 protein.

**Table 2:** Fusion and fission-related genes mRNA expression in vivo.

	SED	EXE	SED + APS	EXE + APS
Mfn-1	1.00	0.60 ± 0.05^*∗*^	1.09 ± 0.15	0.83 ± 0.28^#^
Mfn-2	1.00	0.70 ± 0.15^*∗*^	1.05 ± 0.12	0.85 ± 0.03^#^
Opa-1	1.00	0.90 ± 0.05^*∗*^	0.93 ± 0.15	1.04 ± 0.30
Drp-1	1.00	2.33 ± 0.28^*∗*^	1.10 ± 0.25	1.67 ± 0.13^#^
Fis-1	1.00	1.04 ± 0.47	1.01 ± 0.26	1.14 ± 0.12

Mfn-1: mitofusin 1; Mfn-2: mitofusin 2; Opa-1: optic atrophy 1; Drp-1: dynamin-related protein q; Fis-1: mitochondrial fission 1 protein. ^*∗*^Significant difference between EXE and SED groups (*t*-test; *P* < 0.05). ^#^Significant difference between EXE and EXE + APS groups (*t*-test; *P* < 0.05).

**Table 3:** Fusion and fission-related genes mRNA expression in vitro.

	Control	t-BHP	Control + APS	t-BHP + APS
Mfn-1	1.00	0.88 ± 0.04^*∗*^	1.01 ± 0.12	0.95 ± 0.04^#^
Mfn-2	1.00	0.88 ± 0.15^*∗*^	1.05 ± 0.02^*∗*^	0.99 ± 0.05^#^
Opa-1	1.00	0.97 ± 0.05	1.00 ± 0.13	0.96 ± 0.18
Drp-1	1.00	1.12 ± 0.58	1.13 ± 0.37	1.06 ± 0.16
Fis-1	1.00	1.12 ± 0.49	1.04 ± 0.27	1.13 ± 0.19

Mfn-1: mitofusin 1; Mfn-2: mitofusin 2; Opa-1: optic atrophy 1; Drp-1: dynamin-related protein q; Fis-1: mitochondrial fission 1 protein. ^*∗*^Significant difference between t-BHP and control groups (*t*-test; *P* < 0.05). ^#^Significant difference between t-BHP and t-BHP + APS groups (*t*-test; *P* < 0.05).
