# Supplementary material for: Effects of Astragalus Polysaccharides on Dysfunction of Mitochondrial Dynamics Induced by Oxidative Stress
Source: Oxid Med Cell Longev. 2016 Jan 11;2016:9573291. doi: 10.1155/2016/9573291 (PMC4737051; doi:10.1155/2016/9573291)
Supplement: Supplementary file 1 — Figure S1 shows the result of monosaccharide analysis of Astragalus polysaccharides by Gas Chromatography (GC). Table S1 shows the result of proportional analysis of monosaccharide composition in Astragalus polysaccharides. [file 9573291.f1.doc]

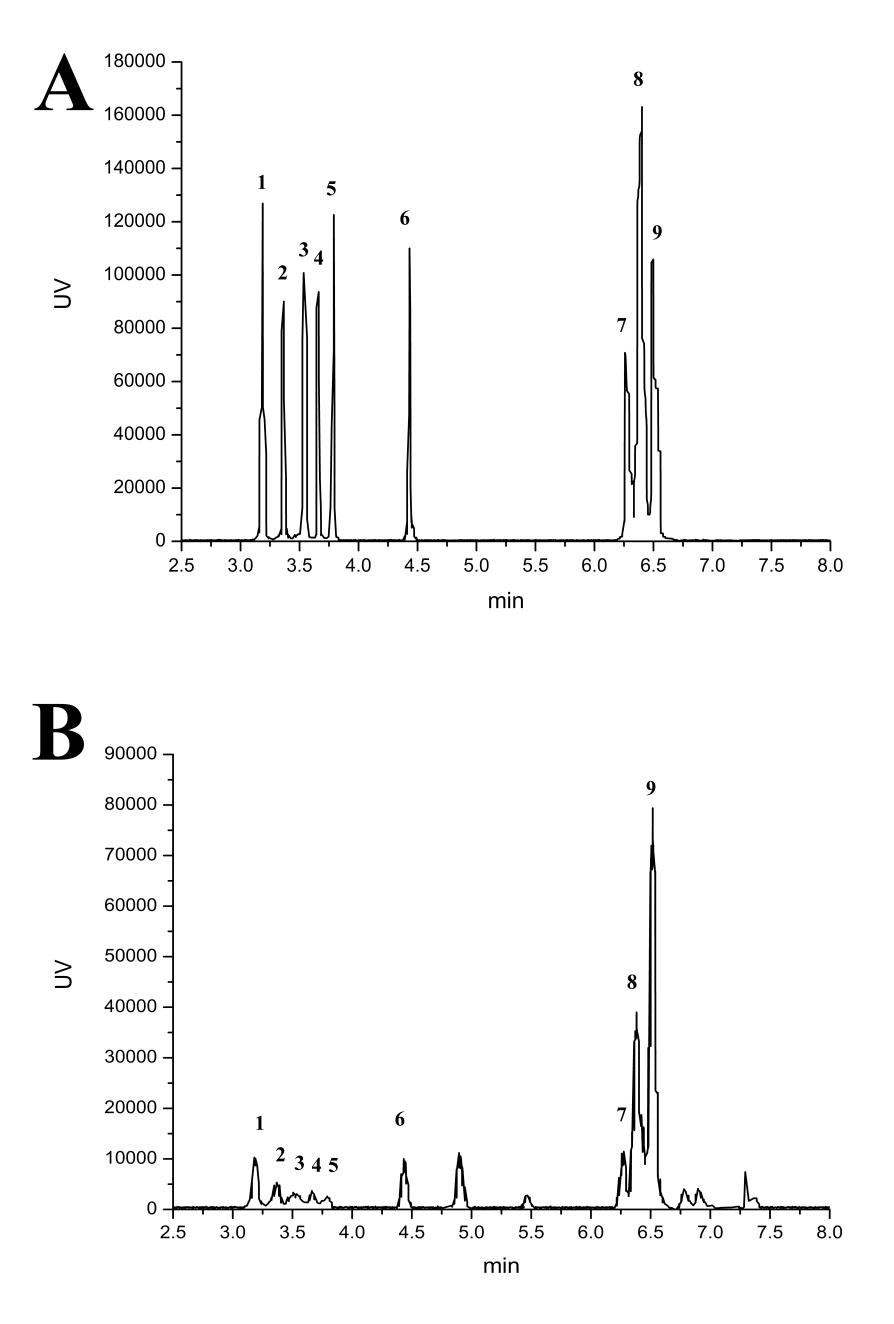


**Figure 1.** Monosaccharide analysis of APS by Gas Chromatography (GC). (A) Chromatogram of standard substances. (B) Chromatogram of APS. 1, L-Rhamnose; 2, D-Xylose; 3, L-Xylose; 4, D-Ribose; 5, L-Ribose; 6, L-Arabinose; 7, D-Galactose; 8, D-Glucose; 9, D-Mannose.

**Table 1. Proportional analysis of monosaccharide composition in APS**

| **Component** | **L-Rha** | **D-Xyl** | **L-Xyl** | **D-Rib** | **L-Rib** | **L-Ara** | **D-Gal** | **D-Glu** | **D-Man** |
| --- | --- | --- | --- | --- | --- | --- | --- | --- | --- |
| **Constituent ratio (%)** | 5.35 | 3.49 | 2.24 | 2.65 | 1.20 | 5.85 | 10.18 | 15.53 | 48.76 |
